# Efficacy and safety of evogliptin in the treatment of type 2 diabetes mellitus in a Brazilian population: a randomized bridging study

**DOI:** 10.1186/s13098-019-0505-z

**Published:** 2019-12-19

**Authors:** Cintia Cercato, Joao Soares Felício, Luis Augusto Tavares Russo, Joao Lindolfo Cunha Borges, Joao Salles, Patricia Muskat, Teresa Bonansea, Antonio Roberto Chacra, Freddy Goldberg Eliaschewitz, Adriana Costa Forti

**Affiliations:** 1 Laboratório de Lípides (LIM 10) do Hospital das Clínicas (HCFMUSP) da Faculdade de Medicina da Universidade de São Paulo, São Paulo, Brasil, 155 Dr Enéas de Carvalho de Aguiar ave., São Paulo, SP 05403-000 Brazil; 2 Hospital Universitário João de Barros Barreto (HUJBB), 4487 Mundurucus st., Belém, PA 66073-005 Brazil; 3 CCBR Brasil - Centro de Pesquisas e Análises Clínicas Ltda., 33 Mena Barreto st., Rio de Janeiro, RJ 22271-100 Brazil; 4 CEM - Clínica de Endocrinologia e Metabologia Ltda., SHIS QI 09 Bloco E1 304/305, Brasília, DF 71625-175 Brazil; 5 Instituto de Pesquisa Clínica Ltda., 503 Marquês de Itú st., São Paulo, SP 01223-001 Brazil; 6 IMA - Instituto de Pesquisa Clínica e Medicina Avançada Ltda., 55 Américo Jacomino sq., São Paulo, SP 05437-010 Brazil; 7 Centro Paulista de Investigação Clínica, 342 Moreira e Costa st., São Paulo, SP 04266-010 Brazil; 8 Centro de Pesquisa Clínica em Diabetes da UNIFESP, 639 Estado de Israel st., São Paulo, SP 04022-001 Brazil; 9 CPCLIN - Centro de Pesquisas Clínicas Ltda., Avenida Angélica 2162, São Paulo, SP 01228-200 Brazil; 10 Centro de Estudos em Diabetes e Hipertensão (CEDH), 2434 Dr Jose Lourenço st., Fortaleza, CE 60115-282 Brazil

**Keywords:** Evogliptin, Dipeptidyl peptidase-4 inhibitor, Type 2 diabetes treatment, Bridging study

## Abstract

**Background:**

Evogliptin (EVO) is a potent and selective dipeptidyl peptidase-4 inhibitor (DPP4i) developed for the treatment of type 2 diabetes mellitus (T2DM). DPP4is are known to exhibit a better glucose-lowering effect in Asians compared to other ethnic groups. Once EVO’s clinical development program was conducted in Asian patients, this bridging study was designed to validate for the Brazilian population the efficacy and safety of the approved dose regimen (once-daily 5.0 mg).

**Methods:**

In this randomized, double-blind, double-dummy, parallel trial, 146 patients with T2DM with inadequate glycemic control on diet and exercise (7.5% ≤ HbA1c ≤ 10.5%) were randomly assigned to a 12-week once-daily treatment with EVO 2.5 mg (N = 35), EVO 5 mg (N = 36), EVO 10 mg (N = 36), or sitagliptin (SITA) 100 mg (N = 39). Absolute changes (Week 12—baseline) in HbA1c, fasting plasma glucose (FPG) and body weight (BW) were obtained. One-sided one sample t test was used to determine if mean HbA1c reduction in each group was < − 0.5% (beneficial metabolic response). An analysis of covariance estimated the change in HbA1c and FPG adjusted by baseline HbA1c, FPG, body mass index (BMI) and study site. Response rates to treatment were also established. No between-group statistical comparisons were planned.

**Results:**

HbA1c mean reductions were − 1.26% (90% CI − 1.7%, − 0.8%), − 1.12% (90% CI − 1.4%, − 0.8%), − 1.29% (90% CI − 1.6%, − 1.0%), and − 1.15% (90% CI − 1.5%, − 0.8%) in groups EVO 2.5 mg, EVO 5 mg, EVO 10 mg, and SITA 100 mg, respectively. FPG levels showed a mean increase of 10.89 mg/dL in group EVO 2.5 mg, with significant mean reductions of − 18.94 mg/dL, − 21.17 mg/dL, and − 39.90 mg/dL in those treated with EVO 5 mg, EVO 10 mg, and SITA 100 mg, respectively. BW showed significant reductions of approximately 1 kg in patients treated with EVO 5 mg, EVO 10 mg, and SITA 100 mg. Mean adjusted reductions of HbA1c and FPG levels confirmed the significant clinical benefit of all study treatments. The clinical benefit of EVO’s “target” dose (5 mg) was confirmed. No safety concerns were identified.

**Conclusions:**

These results validate for the Brazilian population the approved dose regimen of EVO (once-daily 5 mg).

*Trial registration* ClinicalTrials.gov Identifier: NCT02689362 (first posted on 02/23/2016).

## Background

DPP4is are oral antidiabetic agents that inhibit the degradation of incretin hormones, such as glucagon-like peptide (GLP-1) and gastric inhibitory polypeptide (GIP), which are secreted in the in response to food ingestion and promote postprandial insulin secretion [1, 2]. In patients with T2DM, DPP4is reduce the degradation incretin hormones, hence increasing their half-life and promoting their actions (insulin secretion, decrease of gastric emptying rate, and inhibition of glucagon secretion). This class of antidiabetic agents is widely used due to their ease of administration, modest effects on HbA1c, and lack of serious side effects [3].

EVO (DA-1229) is a potent and selective DPP4i developed for the treatment of T2DM [4]. Phase 1 results showed a pharmacokinetic profile that warrants a once-daily dose regimen, with excellent safety profile [5, 6]. Pharmacodynamic evaluations showed that EVO promotes ≥ 80% selective DPP4 inhibition after administration of single ≥ 5 mg doses, with ≥ 80% of the inhibition maintained for more than 36 h in steady state. Moreover, postprandial plasma active GLP-1 concentrations were significantly higher in evogliptin-treated subjects compared to those treated with placebo [6]. In a phase 2 dose-finding study, the treatment of patients with T2DM with EVO 5 mg in monotherapy showed the greatest reduction in HbA1c, comparable those achieved with other approved DPP4is [7].

One multicenter, randomized, double-blind, controlled, phase 3 trial confirmed the efficacy and safety of a once-daily EVO 5 mg monotherapy versus placebo in patients with T2DM inadequately controlled by diet and exercise [8]. Another phase 3 trial determined the non-inferiority and safety of EVO 5 mg compared to sitagliptin in patients with T2DM with inadequate glycemic control under metformin monotherapy (combined therapy) [9]. The results of both pivotal studies supported the regulatory approval of EVO in South Korea.^1^

DPP4is are known to exhibit a better glucose-lowering effect in Asians compared to other ethnic groups due to differences in the pathophysiology of T2DM by ethnic group, with predominance of insulin secretory defect resulting from greater β-cell deficiency in Asians [10–12]. Nonetheless, the approved DPP4is proved to be effective and are used in the same dosages in Asiatic and non-Asiatic countries [12, 13].

EVO’s clinical development program was conducted in South Korea and enrolled Asian patients. This bridging study was therefore designed aiming at validating the efficacy and safety of the data obtained in these studies, which were conducted in Asian population. The confirmation of the clinical benefit of the approved dose regimen (once-daily EVO 5 mg) for the Brazilian population allows the validation of all clinical data obtained previously in Asia. This strategy is in accordance with the International Conference of Harmonisation (ICH) guideline on the acceptability of foreign clinical data for medicines from a well-known drug class which are sensitive to ethnic factors [14].

## Methods

This multicentric, randomized, double-dummy, parallel study was conducted in 10 Brazilian sites from August 2017 to May 2018, and enrolled patients with T2DM with inadequate glycemic control on diet and exercise (Clinicaltrials.gov: NCT02689362). Patients aged 20 to 75 years, with 7.5% ≤ HbA1c ≤ 10.5% at screening, a body mass index (BMI) between 20 kg/m^2^ and 40 kg/m^2^ (limits included), and who had not been on any hypoglycemic agent within 12 weeks prior to screening were considered eligible for the study. Subjects with FPG ≥ 300 mg/dL at screening in the presence of severe signs and/or symptoms of T2DM were excluded. Patients were also excluded in the presence of one or more of the following criteria: New York Heart Association class III or IV congestive heart failure; symptoms of liver and/or gallbladder disease; myocardial infarction, coronary artery bypass surgery, or stroke within 6 months prior to the study screening; history of gastrointestinal tract resection; creatinine clearance (Cockroft–Gault equation) < 60 mL/min; alanine aminotransferase (ALT) and/or aspartate aminotransferase (AST) ≥ 2.5 times the upper limit of normal (ULN); plasma creatine phosphokinase (CPK) ≥ 3 times the ULN; plasma triglycerides > 400 mg/dL; history of significant skin allergy; use of steroids within 3 months prior to screening; use of warfarin, dicumarinic agents, or digoxin; use of CYP3A4 inducers or inhibitors; untreated or decompensated thyroid disease; history of illegal drug or alcohol abuse in the 2 months prior to screening. Pregnant and lactating women were also excluded.

Eligible patients were randomly assigned to a 12-week once-daily treatment with EVO 2.5 mg, EVO 5 mg, EVO 10 mg or SITA 100 mg. All patients were instructed to follow a diet and exercise program during the entire study. HbA1c, FPG and BW were obtained every 4 weeks. The study was approved by the Ethics Committee of each study site, and all patients provided a written informed consent prior to entering the study, which was conducted in compliance with the ethical principles of the Declaration of Helsinki.

The primary endpoint was the change in HbA1c (%) from baseline (screening) to Week 12. Other efficacy endpoints included change from baseline in FPG (mg/dL) and body weight (BW), as well as the response rate (HbA1c < 7.0% or HbA1c < 6.5%) at the end of the study treatment (Week 12). Safety was evaluated by means of adverse events (AEs) reporting and vital signs, physical exam findings, electrocardiogram (EKG), and laboratory tests (hematology, chemistry and urinalysis).

Sample size was estimated to identify a clinically relevant HbA1c mean reduction (≥ 0.5%) within each treatment group, considering a one-sided one sample t test, a standard deviation (SD) of 0.68%, a significance level of 5%, and a dropout rate of 15%. Changes from baseline to Week 12 (mean; 90% CI) in HbA1c and FPG levels, and BW were calculated. Treatment effects on HbA1c and FPG levels adjusted by baseline HbA1c, FPG, BMI and study site were also estimated.

No between-groups comparisons were neither planned nor performed. Descriptive analyses based on bilateral 90% confidence interval (90% CI) were established for each group in order to verify the potential clinical benefit of each individual treatment, aiming to validate the previously approved dosage (once-daily 5 mg) and safety of EVO for the Brazilian population. Absolute changes from baseline to Week 12 in HbA1c, FPG and BW were obtained (mean; bilateral 90% CI). One-sided one sample t test was used to determine if the mean HbA1c reduction in each treatment group was < − 0.5% (meaning a beneficial metabolic response to treatment). An analysis of covariance was performed to estimate the change (Week 12—baseline) in HbA1c and FPG (dependent variables) adjusted by baseline HbA1c, FPG, BMI and study site (independent variables). The response rate was established as the proportion of subjects within each group with HbA1c < 7.0% or HbA1c < 6.5% on Week 12. Efficacy analyses were performed for the intention-to-treat (ITT; primary efficacy analysis) and per protocol (PP) populations.

The incidence of AEs was established for each treatment group. The frequency of clinically relevant (as per the investigator assessment) laboratory tests and EKG changes, as well as vital signs throughout the study were summarized by treatment group. The safety population was used in these analyses.

## Results

**Fig. 1:**
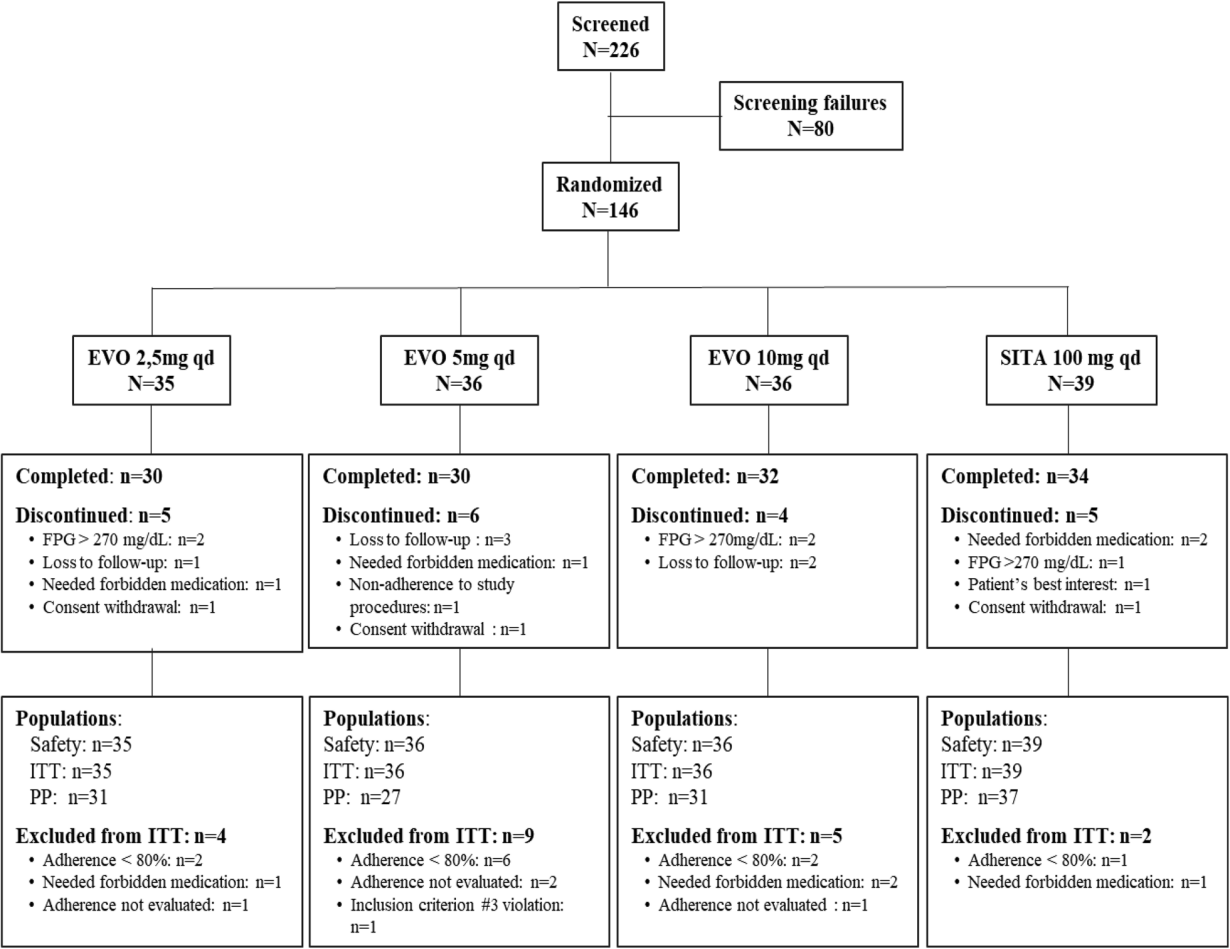
Disposition of subjects in study groups and populations. *EVO* evogliptin, *FPG* fasting plasma glucose, *ITT* intention-to-treat, *PP* per protocol, *qd* once-daily, *SITA* sitagliptin

Of the 226 patients screened, 146 were randomized and received the treatment for which they were allocated (EVO 2.5 mg: N = 35; EVO 5 mg: N = 36; EVO 10 mg: N = 36; SITA 100 mg: N = 39). From the 146 randomized subjects, 126 (86.3%) completed the study. The reasons for study discontinuation and the disposition of participants in the study groups and populations are presented in Fig. 1.

Demographics and baseline clinical characteristics were mostly similar among study groups (Table 1). The exposure to study treatment was also similar in the four treatment groups.

**Table 1:** Demographics and baseline characteristics (ITT population)

	EVO 2.5 mg (N = 35)	EVO 5 mg (N = 36)	EVO 10 mg (N = 36)	SITA 100 mg (N = 39)
Gender, n (%)				
Female	16 (45.7)	19 (52.8)	19 (52.8)	18 (46.2)
Age, years				
Mean (SD)	50.46 (9.33)	53.17 (11.50)	50.11 (9.36)	52.10 (10.41)
BMI, kg/m^2^				
Mean (SD)	29.79 (5.04)	30.56 (4.18)	29.80 (4.71)	30.50 (4.76)
Median	29.3	31.1	29.6	30.6
Min–max	20.8–39.9	22.3–39.6	21.4–39.9	20.9–39.7
T2DM duration, years^a^				
Mean (SD)	2.49 (4.03)	1.12 (2.86)	2.21 (4.42)	1.18 (2.26)
Median	0.6	0.0	0.1	0.2
Min–max	0.0–18.0	0.0–13.6	0.0–21.0	0.0–11.0
HbA1c, %				
Mean (SD)	9.09 (0.99)	8.84 (0.85)	8.95 (0.93)	8.90 (0.94)
Median	9.2	8.8	9.0	9.0
Min–max	7.5–10.5	7.5–10.5	7.5–10.5	7.5–10.5
FPG, mg/dL				
Mean (SD)	168.20 (48.56)	182.19 (42.96)	188.25 (50.12)	193.10 (56.21)
Median	160.0	172.0	185.0	183.0
Min–max	62.0–293.0	109.0–301.0	122.0–336.0	104.0–342.0

*BMI* body mass index, *EVO* evogliptin, *FPG* fasting plasma glucose, *HbA1c* glycated haemoglobin, *ITT* intention-to-treat, *Max* maximum, *Min* minimum, *SD* standard deviation, *SITA* sitagliptin, *T2DM* type 2 diabetes mellitus

^a^Calculated as the number of years between the diagnosis of T2DM and signature of the informed consent

For the ITT population, HbA1c mean reduction (Week 12—baseline) was − 1.26% (90% CI − 1.7%, − 0.8%), − 1.12% (90% CI − 1.4%, − 0.8%), − 1.29% (90% CI − 1.6%, − 1.0%), and − 1.15% (90% CI − 1.5%, − 0.8%) in groups EVO 2.5 mg, EVO 5 mg, EVO 10 mg, and SITA 100 mg, respectively. It is noteworthy to observe that besides being statistically significant, the upper limit of the 90% CI in all study groups was < − 0.5%, the pre-specified limit of clinical significance. Median HbA1c changes were − 0.9%, − 1.1%, − 1.3% e − 1.4% in the groups EVO 2.5 mg, EVO 5 mg, EVO 10 mg, and SITA 100 mg, respectively. FPG levels showed a mean increase of 10.89 mg/dL (90% CI − 5.3 mg/dL, 27.1 mg/dL) in subjects treated with EVO 2.5 mg, whereas significant reductions of − 18.94 mg/dL (90% CI − 31.8 mg/dL, − 6.1 mg/dL), − 21.17 mg/dL (90% CI − 34.2 mg/dL, − 8.2 mg/dL), and − 39.90 mg/dL (90% CI − 55.1 mg/dL, − 24.7 mg/dL) were observed in those treated with EVO 5 mg, EVO 10 mg, and SITA 100 mg, respectively. Median FPG changes were 3.0 mg/dL, − 27 mg/dL, − 20 mg/dL, and − 27 mg/, respectively. BW showed a significant reduction of approximately 1 kg in patients treated with EVO 5 mg (− 1.19 kg; 90% CI − 1.7 kg, − 0.7 kg), EVO 10 mg (− 1.03 kg; 90% CI − 1.8 kg, − 0.3 kg), and SITA 100 mg (− 1.13 kg; 90% CI − 1.8 kg, − 0.4 kg), with no significant change in those treated with EVO 2.5 mg (− 0.08 kg; 90% CI − 0.8 kg, 0.7 kg). Similar results were obtained in the PP population.

**Fig. 2:**
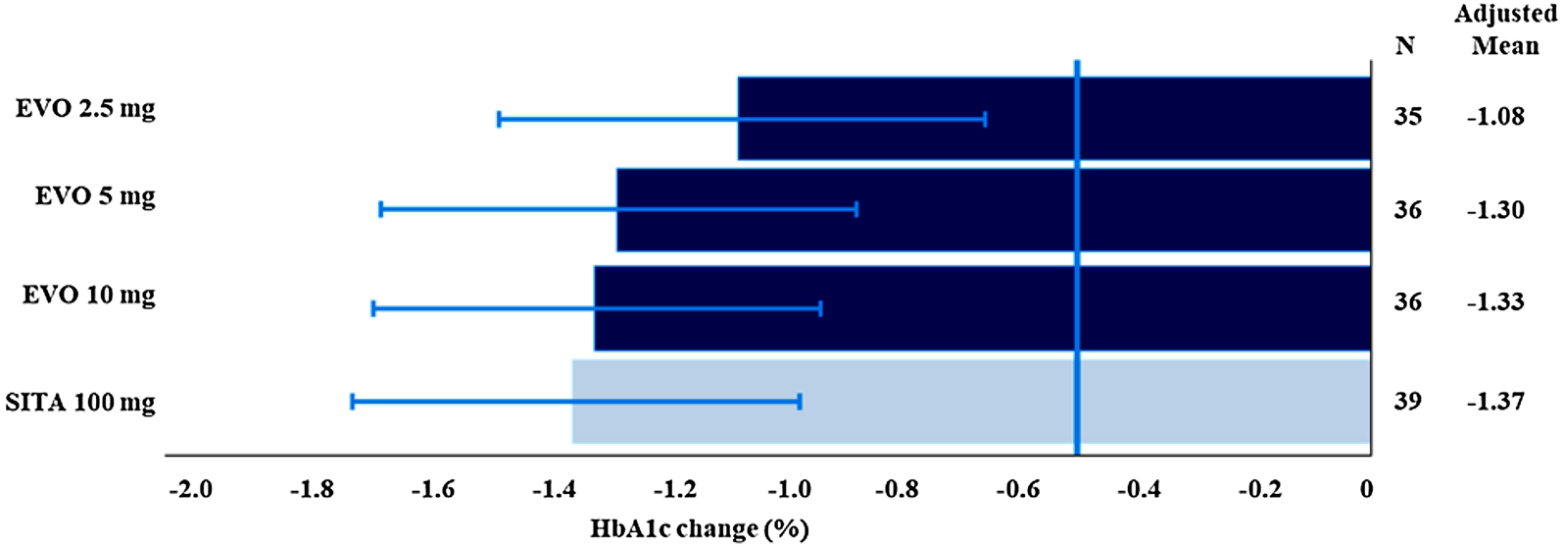
HbA1c change from baseline to Week 12—adjusted mean and 90% CI (ITT population). *EVO* evogliptin, *SITA* sitagliptin

**Fig. 3:**
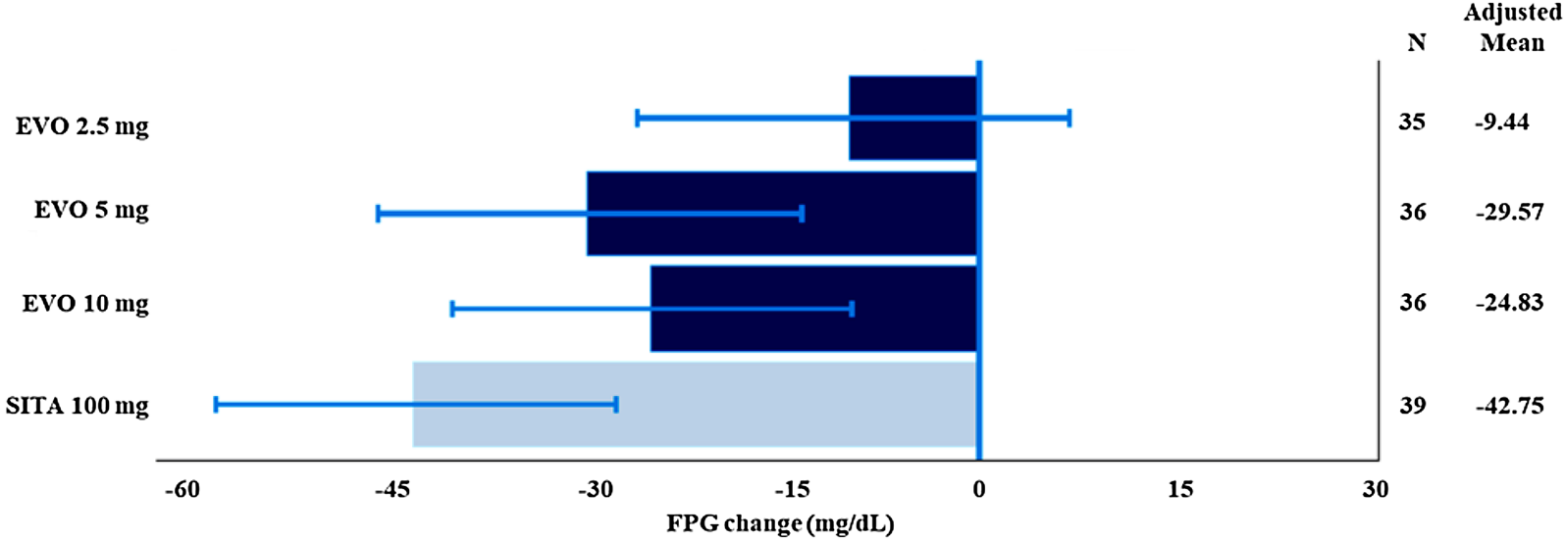
Fasting plasma glucose change from baseline to Week 12—adjusted mean and 90% CI (ITT population). *EVO* evogliptin, *FPG* fasting plasma glucose, *SITA* sitagliptin

Mean reductions of HbA1c and FPG levels at the end of the 12-week study treatment adjusted by baseline HbA1c, FPG, BMI, and study site confirmed the significant clinical benefit of all study treatments, showing upper 90% CIs limits < − 0.5%. These results are illustrated in Figs. 2 and 3 for the ITT population. Similar results were obtained in the PP population.

For the ITT population, the response rate, defined as the proportion of patients achieving HbA1c < 7.0% at the end of the study treatment, was 22.9% (90% CI 11.9%, 37.5%) in the group EVO 2.5 mg, 30.6% (90% CI 18.2%, 45.5%) in the group EVO 5 mg, 27.8% (90% CI 15.9%, 42.6%) in the group EVO 10 mg, and 41.0% (90% CI 27.7%, 55.4%) in the group SITA 100 mg. The proportion of patients with HbA1c < 6.5% at the end of the study was 14.3% (90% CI 5.8%, 27.7%), 5.6% (90% CI 1.0%, 16.5%), 19.4% (90% CI 9.5%, 33.4%), and 18.0% (90% CI 8.7%, 31.1%) in subjects treated with EVO 2.5 mg, EVO 5 mg, EVO 10 mg, and SITA 100 mg, respectively. Similar results were obtained in the PP population.

Treatment-emergent AEs were reported by 88 (60.3%) subjects of the safety population (N = 146). The distribution of the reported AEs per study group is summarized in Table 2. AEs reported by ≥ 5% of the subjects in at least one study group are presented in Table 3.

**Table 2:** Adverse events summary (safety population)

	EVO 2.5 mg (N = 35)	EVO 5 mg (N = 36)	EVO 10 mg (N = 36)	SITA 100 mg (N = 39)
Subjects with at least 1 AE				
N (%)	23 (65.7)	24 (66.7)	21 (58.3)	20 (51.3)
90% CI	50.5 78.9	51.7 79.5	43.3 72.3	37.1 65.3
Discontinuations due to AEs				
N (%)	2 (5.7)	1 (2.8)	0 (0.0)	1 (2.6)
Reported AEs				
N	40	49	41	39
AEs related^a^ to study treatment				
N	0	4	0	7
SAEs				
N	1	1	1	1
Deaths				
N	0	0	0	0

*AE* adverse event, *90% CI* 90% confidence interval, *EVO* evogliptin, *SAE* serious adverse event, *SITA* sitagliptin

^a^Possibly or probably related to the study treatment

**Table 3:** Adverse events reported by ≥ 5% of the patients of any study group (safety population)

Preferred term (MedDra)	EVO 2.5 mg (N = 35)	EVO 5 mg (N = 36)	EVO 10 mg (N = 36)	SITA 100 mg (N = 39)
Headache	0 (0.0)	5 (13.9)	1 (2.8)	1 (2.6)
GGT elevated	4 (11.4)	0 (0.0)	0 (0.0)	0 (0.0)
Hypertension	3 (8.6)	2 (5.6)	0 (0.0)	3 (7.7)
Diarrhea	3 (8.6)	1 (2.8)	1 (2.8)	2 (5.1)
Flu-like symptoms	1 (2.9)	2 (5.6)	3 (8.3)	3 (7.7)
Urinary tract infection	1 (2.9)	2 (5.6)	3 (8.3)	2 (5.1)
T2DM with inadequate control	2 (5.7)	1 (2.8)	0 (0.0)	1 (2.6)
Dizziness	0 (0.0)	1 (2.8)	2 (5.6)	1 (2.6)
Eosinophilia	0 (0.0)	2 (5.6)	0 (0.0)	1 (2.6)
Abdominal pain	1 (2.9)	1 (2.8)	2 (5.6)	0 (0.0)
Dyslipidemia	1 (2.9)	0 (0.0)	2 (5.6)	0 (0.0)
Back pain	0 (0.0)	1 (2.8)	2 (5.6)	0 (0.0)
Upper airway infection	1 (2.9)	2 (5.6)	0 (0.0)	0 (0.0)
Dyspepsia	0 (0.0)	0 (0.0)	2 (5.6)	0 (0.0)
Nausea	1 (2.9)	1 (2.8)	1 (2.8)	2 (5.1)
Hyperglycemia	1 (2.9)	0 (0.0)	0 (0.0)	2 (5.1)

*EVO* evogliptin, *GGT* gamma-glutamyl transferase, *T2DM* type 2 diabetes mellitus

## Discussion

The primary efficacy analysis showed statistically and clinically significant reductions of HbA1c levels, indicating the clinical benefit of all study treatments, including the “target” group treated with EVO 5 mg, in which the mean HbA1c level absolute change was − 1.12% (90% CI − 1.4%, − 0.8%). The observed reductions of HbA1c levels observed at the end of the 12-week study treatment compared to baseline values were greater than 1% in all study groups; these reductions are greater than the reported reductions of HbA1c previously reported for DPP4i in monotherapy (which vary mostly from 0.5 to 0.8%) [3, 15–20], possibly due to the high HbA1c levels at baseline. Similarly to the results of a phase II study that aimed to determine the optimal dose of EVO in a Korean population [7], our results failed to show a dose-dependent reduction of HbA1c levels. Likewise, all studied dosages (2.5, 5 and 10 mg) resulted in significant and relevant reductions of HbA1c levels, and the optimal dosage (5 mg) was determined through the evaluation of all endpoints in that population of patients.

FPG levels also showed significant reductions in subjects treated with EVO 5 and 10 mg, as well as in those treated with SITA 100 mg, but not in those treated with EVO 2.5 mg. The mean change of FPG associated to the daily monotherapy with SITA 100 mg [− 39.9 mg/dL (90% CI − 55.1 mg/dL, − 24.7 mg/dL)] was similar to that observed in previous studies [15], confirming the internal validity of our results. The lack of response on FPG levels observed in subjects treated with EVO 2.5 mg may be explained by the small activity of DPP4is on fasting glycaemia, once these agents are known to mainly promote the reduction of postprandial glycaemia [21]. With exception of the patients treated with EVO 2.5 mg, the reduction of BW observed is in accordance to the expected for DPP4is [22]. The observed response rates also correspond to those observed with DPP4is in monotherapy [15].

This study was not designed to allow between-group statistical comparisons. A group treated with SITA 100 mg was included to enable the validation of our results once the question “Is the studied population responsive to an DPP4i of proved efficacy?” can be answered by the observation of the results of this group. The validation of the efficacy of daily administration of EVO 5 mg in the studied population is supported by the clinical benefit associated to this dose regimen in terms of absolute reduction of HbA1c and FPG levels. The adjusted reductions of HbA1c and FPG levels confirmed these findings.

The sample size was calculated in order to allow the identification of clinically relevant HbA1c reductions (≥ 0.5%) within each treatment group and did not allow between-group comparisons. Despite the fact that between-group comparisons were beyond this bridging study objectives. This might be considered a limitation of our study. Despite the fact that this study was not designed to allow statistical between-groups comparisons, it is noticeable that (a) the reduction of HbA1c showed by patients treated with EVO 5 mg was similar to that observed with EVO 2.5 mg, while only the treatment with EVO 5 mg was associated with FPG and BW significant reduction, and (b) compared to the treatment with EVO 10 mg, the 12-week daily administration of EVO 5 mg showed similar reductions of HbA1c, FPG and BW, being the lower dosage preferred over the higher. Therefore, our results validate the efficacy of the previously approved dose regimen of EVO (once-daily 5 mg) to the Brazilian population.

Once-daily treatment with EVO 5 mg was safe and well tolerated. The most frequently reported AEs were similar to those observed with other DPP4is.

## Conclusions

The results of this bridging study validate for the Brazilian population the previously approved dose regimen of EVO (once-daily 5 mg), hence validate the results of the studies conducted in Korea during its clinical development program for local approval in Brazil.

## Data Availability

The data that support the findings of this study are available from Eurofarma Laboratórios S.A. (study sponsor), but restrictions apply to the availability of these data, which were used under license for the current study, and so are not publicly available. Data are however available from the corresponding author upon reasonable request and with permission of Eurofarma Laboratórios S.A.

## Abbreviations

AE: adverse event
ALT: alanine aminotransferase
AST: aspartate aminotransferase
BW: body weight
90% CI: 90% confidence interval
DPP4i: dipeptidyl peptidase-4 inhibitor
EKG: electrocardiogram
EVO: evogliptin
FPG: fasting plasma glucose
GIP: gastric inhibitory polypeptide
GLP-1: glucagon-like peptide 1
HbA1c: glycated hemoglobin
ITT: intention-to-treat
PP: per protocol
qd: once-daily
SD: standard deviation
SITA: sitagliptin
T2DM: type 2 diabetes mellitus
ULN: upper limit of normal

## Footnotes

1 Suganon^®^—Dong-A ST.
